# Patho-radiological concordance in brain tumors - A retrospective study

**DOI:** 10.6026/973206300212196

**Published:** 2025-07-31

**Authors:** Tarang Patel, Virendrakumar Meena, Manisha Jain, Kesha Rachani, Krupal Joshi, Deepa Shukla

**Affiliations:** 1 Department of Pathology, All India Institute of Medical Sciences(AIIMS), Rajkot, Gujarat, India; 2 Department of Radiodiagnosis, Geetanjali Medical College & Hospital, Udaipur, Rajasthan, India; 3 Department of Pathology, Geetanjali Medical College & Hospital, Udaipur, Rajasthan, India; 4 Department of Community and Family Medicine, All India Institute of Medical Sciences(AIIMS), Rajkot, Gujarat, India; 5 Department of AYUSH, All India Institute of Medical Sciences(AIIMS), Jodhpur, Rajasthan, India

**Keywords:** Radiological concordance, magnetic resonance imaging, astrocytoma, glioblastoma, meningioma

## Abstract

This study analyzed 102 brain tumor cases using MRI imaging and pathological data. The cerebrum was the most common affected site, with meningioma being the most common. The concordance between MRI and biopsy reports was mild to moderate. Data shows that MRI imaging may increase diagnostic accuracy of brain tumors and should be routinely performed in all suspicious cases. Correlation of MRI findings can help rule out other mimickers of intracranial mass.

## Background:

Despite the availability of advanced imaging techniques, histological inspection remains the gold standard, as most brain tumors exhibit distinct histomorphological features reflecting their heterogeneity [1]. Neuroradiology, now a highly advanced field in specialized centers, has significantly improved diagnostic yield with the advent of Magnetic Resonance Imaging (MRI), including perfusion imaging and diffusion-weighted imaging [2]. Pathologists often rely on radiological features alongside clinical and per-operative findings to enhance the interpretation of Central Nervous System (CNS) malignancies, yet despite these advancements, accurate diagnosis remains challenging for neurosurgeons and pathologists in rural or primary care settings [3]. Radiological findings of suspected brain tumors aid pathologists in narrowing differential diagnoses during final histopathological assessment, with a location-based approach further enhancing the accuracy of CNS malignancy diagnosis [4]. According to the latest WHO classification, CNS tumors are categorized into various subgroups, including glial tumors such as astrocytoma, ependymoma, glioblastoma and oligodendroglioma, as well as non-glial neoplasms like tumors of the sellar region, choroid plexus, pineal gland, meninges, nerve sheath, embryonal origin, hematopoietic neoplasms and metastatic lesions [5]. Sophisticated, contemporary non-invasive and invasive radiological studies, intraoperative squash cytology, post-operative biopsy and tumor histology are all necessary for an accurate diagnosis of CNS neoplasms [6]. Therefore, it is of interest to describe innovative approaches combining these diagnostic tools to enhance the precision and equity of CNS tumor diagnosis.

## Materials and Methods:

## Study participants:

The retrospective study was conducted in the Departments of Pathology and Radiodiagnosis. It included various paraffin blocks, histopathology slides, clinicopathological details, and radiological details including MRI findings. The study included 102 cases of brain tumors. Cases were included from January 2019 to October 2021. Demographic details and clinical data were retrospectively collected from known cases of brain tumors admitted and treated in tertiary cancer centers.

## Inclusion criteria:

Cases suspicious of brain tumors and then diagnosis confirmed on histopathological or frozen specimen examination, cases with complete MRI findings & cases treated in the same institute were included in the study.

## Exclusion criteria:

Cases without histopathological confirmation and cases with incomplete radiological profiles were excluded from the study. Pure intraspinal masses were excluded from the study.

## Biopsy procedure:

In required cases, Intraoperative Frozen sections using stereotactic biopsy were sent to the Histopathology department for intraoperative diagnostic consultation. Squash cytology smears were also prepared to assist in the diagnosis of neuropathology. This was followed by routine histopathological diagnosis using Hematoxylin and Eosin staining. All findings were reported by an expert histopathologist.

## Imaging procedure:

MR brain imaging was done with a 3-Tesla scanner (SIGNA ARCHITECT) by using the following sequences: volumetric 3D T1 weighted sequence, T2 weighted sequence, 3D FLAIR (Fluid-attenuated inversion recovery) sequence, Diffusion Weighted sequence, Post contrast 3D T1 weighted sequence Susceptibility weighted imaging (SWI), ASL sequence and Multi voxel spectroscopy sequence. Radio imaging (MRI) findings of all cases were retrieved retrospectively and histopathology (HP) diagnoses were correlated with corresponding MRI diagnoses. If MRI diagnosis was compatible with HP diagnosis, it was categorized as "concordance". If an MRI impression suggests more than one diagnosis including the final HP diagnosis, it is considered as "partial concordance". MRI diagnoses not compatible with HP diagnosis were considered "non-concordance".

## Results:

A total of 102 cases were retrieved and diagnosed as CNS tumors on histopathology. Additionally, five patients diagnosed with CNS tumors on radiology emerged as negative for tumors on microscopy. Eight cases clinically suspicious for CNS tumors were negative on both radiology and histopathology. Patient's age ranges from 11 to 72 years. 57 were male patients and 45 were female patients. MRI findings were retrieved from all patients. Microscopic diagnosis was confirmed in all 102 cases either during routine histopathological examination or while examining intraoperative squash cytology or tissue biopsy. Detail of all 102 cases as per their site, age, gender and histopathological diagnosis. Among all cases, cerebrum was the most common site involved, which included 29 cases of the frontal lobe, 14 cases of the parietal lobe, nine cases of the temporal lobe, temporo-parietal in six cases, fronto-temporal in two cases, parieto-occipital in two cases, fronto-parietal in one case and fronto-parieto-temporal in one case. Other cases from posterior fossa (six), cerebellopontine(CP) angle (nine), suprasellar (five), parasagittal (four), intraventricular (four), cerebellar (four), corpus callosum (two), pure intradural (two) and each case of thalamus and insula. Out of a total of 103 cases, the most common tumor type was Meningioma consisting of 23 cases (20 of grade I, three of grade II), followed by Glioblastoma grade 21 cases), Astrocytoma (12 cases: two of grade I, five of grade II, five of grade III) and Oligodendroglioma (eight cases: 1 of grade II, seven of grade III). Other histopathological diagnoses were Schwannoma (seven cases), Ependymoma (five cases: three cases of grade III & two cases of grade II), Medulloblastoma (five cases) and Pituitary adenoma (four cases). Other less frequent Histopathology (HP) diagnosis were Hemangioblastoma(two), Metastatic carcinoma(two), Ganglioglioma(two) and Anaplastic ganglioglioma(one) cases. Two cases of glioma could not be classified further and were diagnosed as each case of Glioma grade II & Glioma grade III. Other rare diagnoses include each case of Colloid cyst of the ventricle, Fungal infection, Central Neurocytoma, Cavernous hemangioma, Oligoastrocytoma- low grade, Intraventricular ganglioneuroblastoma and Craniopharyngioma.

Of 102 brain tumor cases, meningioma was the most common (23 cases, 22%), with 74% (17) concordance, 13% (3) partial concordance, and 13% (3) non-concordance. Glioblastoma (22 cases) showed 77% (17) concordance and 23% (5) non-concordance. Low-grade glioma (7 cases) had 44% (3) concordance, 28% (2) partial concordance and 28% (2) non-concordance. Anaplastic astrocytoma (5 cases) showed 20% (1) concordance and 80% (4) non-concordance. Ependymoma (5 cases) had 60% (3) concordance and 40% (2) partial concordance. Oligodendroglioma (8 cases) showed 25% (2) concordance and 75% (6) partial concordance. Medulloblastoma (5 cases) had 40% (2) concordance, 40% (2) partial concordance, and 20% (1) non-concordance. Schwannoma (7 cases) showed 43% (3) concordance and 57% (4) partial concordance. Pituitary adenoma (4 cases) had 25% (1) concordance and 75% (3) partial concordance. Hemangioblastoma (2 cases) had one partial concordance and one non-concordance. Metastatic carcinoma (2 cases) had one concordance and one partial concordance. Ganglioglioma (2 cases) was non-concordant. Single cases of low-grade glioma and cavernous hemangioma were concordant; choroid plexus papilloma, central neurocytoma, craniopharyngioma, and low-grade oligoastrocytoma were partially concordant; high-grade glioma, colloid cyst, anaplastic ganglioglioma, and ganglioneuroblastoma were non-concordant (Figures 1 and 2 - see PDF) depict selected cases. Concordance agreement between conventional MRI and histopathological diagnosis was calculated by Cohen's kappa (κ) measurement utilizing IBM SPSS (Statistical Package for Social Sciences) 27 version. Results of κ value of 0.255 indicate mild to moderate agreement and concordance between MRI diagnosis and histopathology reports. Furthermore, since p is 0.002, our kappa (κ) coefficient is statistically significantly different from zero.

## Discussion:

CNS tumors are rare and they constitute about 2% of all malignancies in India. The incidence rate of CNS tumors in India is about 5-10 per 1 lac human population. CNS tumors are the second most common cancer after leukemia in children. Though brain tumors are heterogeneous, they can be diagnosed on microscopic examination, utilizing their characteristic histopathological features [7]. According to the GLOBOCAN Project (2012), CNS tumors accounted for 1.6% of the global incidence and 2.6% of mortality, with a five-year prevalence rate of 15.2 per 100,000 populations [8]. Accurate diagnosis in patients with brain lesions is essential for selecting appropriate therapy, avoiding unnecessary brain surgery and preventing delays in treatment initiation. Literature on diagnostic accuracy has demonstrated that MRI outperforms contrast-enhanced CT in detecting brain metastases [9]. Conventional MRI technology primarily offers anatomical & structural details about the relationship between a brain tumor and adjacent tissues, aiding in distinguishing brain tumors from other CNS pathologies [10]. Glioblastoma, IDH (Isocitrate Dehydrogenase)-wild type, Grade 4, typically shows necrosis and/or microvascular proliferation. IHC-wild type astrocytoma Grade 2 or 3 should be diagnosed as glioblastoma even in absence of characteristic histopathological findings if any one molecular alteration is present out of EGFR amplification/TERTp mutation/+7/-10 [11]. Diffuse astrocytoma, IDH mutant can be WHO grade 2 or 3, based on mitotic count. Foci of necrosis and/or microvascular proliferation are consistent with Astrocytoma, IDH mutant, Grade 4 [12].

Low-grade gliomas (WHO Grade 1) include pediatric-type diffuse gliomas and circumscribed astrocytic gliomas, classified by MYB/MYBL1 or MAPK pathway alterations. Pilocytic astrocytoma is the most common Grade 1 glioma, while oligodendrogliomas are IDH-mutant with 1p/19q co-deletion and show fried egg appearance with chicken-wire vasculature [13]. Medulloblastomas are the most common embryonal tumors, classified into four histologic and molecular subtypes based on WNT & SHH. Meningiomas have 15 histologic variants, with NF2-associated types usually of higher grade and convex location. Ependymomas are classified by histology, location and molecular features (ZFTA/YAP1 fusions) [14]. Other Grade 1 tumors include choroid plexus papilloma, hemangioblastoma, schwannoma and ganglioglioma (MAPK-altered), with anaplastic variants showing high mitotic activity. Sellar tumors include pituitary adenomas (neuroendocrine) and craniopharyngiomas (adamantinomatous/papillary). Metastatic carcinomas mimic the histology of their primary sites [15]. Glioblastomas appear hypointense on T1W and hyperintense on T2W/FLAIR with characteristic ring enhancement, well-defined edema, restricted diffusion and increased perfusion due to high vascularity [16]. Grade 2/3 (anaplastic) astrocytomas are hypointense on T1W, hyperintense on T2W and show a T2-FLAIR mismatch sign (hypointense core with hyperintense rim), patchy enhancement, blooming on GRE, and elevated cerebral blood volume [17]. Grade 1 astrocytomas also show T2-FLAIR mismatch and GRE blooming but lack contrast enhancement and perfusion elevation; however they may contain cystic components or calcifications [18]. Meningiomas are extra-axial, isointense to mildly hypointense on T1W, hyperintense on T2W and show strong, homogeneous enhancement [19]. Central neurocytomas, typically near the foramen of Monro, appear isointense on T1W, iso- to hyperintense on T2W with bubbly cystic areas, calcifications, and restricted diffusion in solid parts [20]. Oligodendrogliomas are T1 hypointense, T2 hyperintense, with calcifications appearing as blooming on SWI [21]. Medulloblastomas arise in the cerebellar vermis, are T1 hypointense, T2 iso- to hyperintense, enhance post-contrast and show restricted diffusion due to hypercellularity [22].

Choroid plexus papillomas are T1/T2 iso- to hyperintense; hemangioblastomas show T2 hyperintense nodules; ependymomas are T1 iso-/hypointense, T2 hyperintense with GRE blooming; metastases show variable enhancement with diffusion restriction; schwannomas are T1 iso-/hypointense, T2 heterogeneously hyperintense with cysts [15]. Cavernous hemangiomas show "popcorn" appearance with hypointense hemosiderin rim [23]. Gliosarcoma appears T1W hypointense and T2W heterogeneous signal due to hemorrhage and necrosis [24]. Craniopharyngiomas are cystic/calcified with T2 hyperintensity and a lipid-lactate peak on MR spectroscopy, while high-grade gliomas, gangliogliomas, pituitary adenomas, fungal abscesses, and others show variable features with diffusion restriction, enhancement, and perfusion aiding diagnosis [19]. A study by Bhattacharya *et al.* [25] showed meningioma (54.76%. 23/42) as the most common CNS tumor followed by neuroepithelial tumors (38.09%), which differ from our study results. Mourya *et al.* [26] study depicted neuroepithelial tumor as the most common tumor type (46.08%, 53/115), followed by meningioma (27.82%). The neuroepithelial tumor was the most common tumor (44.5%, 101/227) in a study by Mohammed *et al.* [27], followed by meningioma (32.25%, 70/217). With similar results, our study presented neuroepithelial tumor as the most common tumor type (55.9%, 57/102), followed by meningioma (22.5%, 23/102). A study by Abdulkasim *et al.* [9] suggested concordance between conventional MRI diagnosis and final histopathological diagnosis was fair having κ value of 0.38. This result is comparable to our study having κ value of 0.255 and a significant p-value of 0.002.

## Study limitations:

Limitations of the study include a lack of comparison of radiological findings with histological subtypes. In the future, further studies may conduct to correlate MRI findings of brain tumors with their molecular characterization.

## Conclusion:

Brain tumors are challenging to diagnose accurately using routine histopathology or frozen sections, even for experienced pathologists. MRI findings often align with final diagnoses and should guide pathologists in narrowing differential diagnoses. Collaboration with onco-radiologists and special investigations enhance diagnostic accuracy and further patient management.
